# D2 lymphadenectomy is not only safe but necessary in the era of neoadjuvant chemotherapy

**DOI:** 10.1186/1477-7819-11-31

**Published:** 2013-02-02

**Authors:** Shailesh V Shrikhande, Savio G Barreto, Sanjay D Talole, Kumar Vinchurkar, Somashekar Annaiah, Kunal Suradkar, Shaesta Mehta, Mahesh Goel

**Affiliations:** 1 Department of Gastrointestinal and Hepato-Pancreato-Biliary Surgical Oncology, Tata Memorial Hospital, Mumbai, India; 2 Department of Biostatistics and Epidemiology, Tata Memorial Hospital, Mumbai, India; 3 Department of Digestive Diseases and Clinical Nutrition, Tata Memorial Hospital, Mumbai, India

**Keywords:** D2 lymphadenectomy, Gastrectomy, Gastric cancer, Morbidity, Mortality, Neoadjuvant chemotherapy

## Abstract

**Background:**

Patients with locally advanced resectable gastric cancers are increasingly offered neoadjuvant chemotherapy (NACT) following the MAGIC and REAL-2 trials. However, information on the toxicity of NACT, its effects on perioperative surgical outcomes and tumor response is not widely reported in literature.

**Methods:**

Analysis of a prospective database of gastric cancer patients undergoing radical D2 gastrectomy over 2 years was performed. Chemotherapy-related toxicity, perioperative outcomes and histopathological responses to NACT were analyzed. The data is presented and compared to a cohort of patients undergoing upfront surgery in the same time period.

**Results:**

In this study, 139 patients (42 female and 97 male patients, median age 53 years) with gastric adenocarcinoma received NACT. Chemotherapy-related toxicity was noted in 32% of patients. Of the 139 patients, 129 underwent gastrectomy with D2 lymphadenectomy, with 12% morbidity and no mortality. Major pathological response of primary tumor was noted in 22 patients (17%). Of these 22 patients, lymph node metastases were noted in 12 patients. The median blood loss and lymph node yield was not significantly different to the 62 patients who underwent upfront surgery. Patients who underwent upfront surgery were older (58 vs. 52 years, *P* <0.02), had a higher number of distal cancers (63% vs. 82%, *P* <0.015) and a longer hospital stay (11 vs. 9 days, *P* <0.001).

**Conclusions:**

Perioperative outcomes of gastrectomy with D2 lymphadenectomy for locally advanced, resectable gastric cancer were not influenced by NACT. The number of lymph nodes harvested was unaltered by NACT but, more pertinently, metastases to lymph nodes were noted even in patients with a major pathological response of the primary tumor. D2 lymphadenectomy should be performed in all patients irrespective of the degree of response to NACT.

## Background

Gastric cancer is an aggressive disease with a poor survival rate even in surgically resected patients owing to locoregional recurrence [1,2]. In India, gastric cancer is relatively common and is the second most common cause of cancer-related deaths among men and women [3], with 5-year survival rates of just 27% for surgically resected patients [4]. Morbidity and mortality rates following gastric cancer surgery vary around the world depending on the extent of gastric resection and lymphadenectomy [5-8]. While mortality rates have decreased, there continues to be considerable morbidity after surgery [9]. In 2006, Cunningham *et al*. [10] published a landmark trial demonstrating a significant improvement in 5-year survival rates with perioperative chemotherapy for patients with oesphagogastric cancer compared to surgery alone (36% vs. 23%, *P* = 0.009). This trial was followed by another randomized controlled trial in France [11], demonstrating similar improvement in 5-year survival (38% vs. 24%, *P* = 0.02) with the use of neoadjuvant chemotherapy (NACT) as part of perioperative chemotherapy for oesphagogastric cancer. Following these trials [10,11], perioperative chemotherapy for gastric cancer has been increasingly practiced around the world [9].

**Table 1:** Details of the neoadjuvant chemotherapy (NACT) regimens administered and associated toxicity

**Regimen**	**Total number of patients (n = 139)**	**Number of patients who completed the NACT (n = 133)**^**a**^	**Toxicity grade**
EOX3	87	83 (1 patient received 2 additional cycles)	*Tolerated toxicities*
			Grade I vomiting (5 patients)
			Grade I diarrhea (1 patient)
			Grade I vomiting and diarrhea (2 patients)
			Grade I skin and hair toxicity (1 patient)
			Grade I neutropenia (1 patient)
			Grade II vomiting (3 patients)
			Grade II diarrhea (1 patient)
			Grade II vomiting and diarrhea (1 patient)
			Grade III hand-foot syndrome (1 patient)
			Grade IV diarrhea (1 patient)
			Grade IV thrombocytopenia (2 patients)
			*Toxicities resulting in alterations in regimen*
			Grade I diarrhea, and on and off febrile illness (1 patient, resulted in reduced cycles)
			Grade III fatigue (1 patient, resulted in reduced cycles)
			Grade III diarrhea (1 patient, resulted in reduced cycles)
			Grade IV vomiting and diarrhea (1 patient, resulted in reduced cycles)
			Grade III vomiting (1 patient, protocol changed to EOF after 1 cycle)
ECF3	24	23 (1 patient received an additional cycle of docetaxel and oxaliplatin)	Grade I diarrhea (2 patients)
			Grade I vomiting (1 patient)
			Grade II vomiting and diarrhea (1 patient)
			Grade IV mucositis (2 patients)
			Grade IV diarrhea (1 patient)
			Grade IV vomiting and diarrhea (1 patient)
			*Toxicities resulting in alterations in regimen*
			Grade IV diarrhea (1 patient, resulted in reduced cycles)
EOF3	9	8	Grade I vomiting (1 patient)
			Grade I diarrhea (2 patients)
			*Toxicities resulting in alterations in regimen*
			Grade IV mucositis (1 patient, resulted in 25% dose reduction and reduced cycles)
			Grade III febrile neutropenia (1 patient, resulted in 20% dose reduction)
CAPOX3	7	7	Grade 1 nausea, vomiting (2 patients)
EO3	1	1	
DCF	6	6 (3 patients received 3 cycles each and 1 patient each received 4, 5 and 6 cycles, respectively)	Anemia after 1 cycle and grade 1 diarrhea after 2 cycles (1 patient)
			Grade II febrile neutropenia (1 patient)
			Grade III diarrhea (1 patient)
			1 patient developed chicken pox while on chemotherapy
ECX3	5	5	*Toxicities resulting in alterations in regimen*
			Grade 1 nausea and vomiting (1 patient, protocol changed to ECF after 1 cycle)

^a^Six patients received reduced cycles due to toxicity. E, epirubicin 50 mg/m^2^ (day 1); C, cisplatin 60 mg/m^2^ (day 1); F, 5-FU 650 mg/m^2^ (days 1 to 5); O, oxaliplatin 130 mg/m^2^ (day 1); X, capecitabine 1000 mg/m^2^ twice daily (days 1 to 14); D, docetaxel 50 mg/m^2^ (day 1).

**Table 2:** Perioperative details comparing patients who underwent radical resections following NACT (n = 126) from group 1 with patients who underwent upfront surgery (n = 62) from group 2

	**Resected patients from group 1 (n = 126)**	**Resected patients from group 2 (n = 62)**	
Median age in years (range)	53 (22 to 77)	58 (28 to 82)	*P* <0.02
Sex (M:F)	87:39 (69%:31%)	48:14 (77%:23%)	*P* <0.33
*Surgeries*			
Total gastrectomy	17	6	*P* <0.015
Proximal gastrectomy	30	5	
Subtotal gastrectomy	79	51	
Median blood loss in ml (range)	500 (150 to 1700)	400 (50 to 900)	*P* <0.24
Median units of blood transfused (range)	0 (0 to 2)	0 (0 to 2) 0.88	*P* <0.88
Median lymph nodes (range)	16 (1 to 53)	18 (3 to 39)	*P* <0.25
Morbidity rate	12% (15)	22.6% (14)	*P* <0.056
Mortality rate	0% (0)	0% (0)	-
Median duration of hospital stay in days (range)	9 (6 to 28)	11 (6 to 43)	*P* <0.001

**Table 3:** Histopathological data comparing patients who underwent radical resections following NACT (n = 126) from group 1 with patients who underwent upfront surgery (n = 62) from group 2

	**Resected patients from group 1 (n = 126)**	**Resected patients from group 2 (n = 62)**
*Final pathology*		
Carcinoma *in situ*	-	1 (2%)
Well-differentiated adenocarcinoma	4 (3%)	5 (8%)
Moderately-differentiated adenocarcinoma	27 (21%)	17 (27%)
Poorly differentiated adenocarcinoma	57 (45%)	33 (53%)
Signet ring cell adenocarcinoma	16 (13%)	6 (10%)
Microscopic foci of adenocarcinoma	7 (6%)	-
No residual tumor	15 (12%)	-
*T-stage*		
T1	9	10
T2	26	14
T3	54	18
T4	20	20
Perineural invasion present	22 (17%)	9 (14.5%)
Lymphovascular invasion present	45 (36%)	36 (58%)
Median number of lymph nodes harvested (range)	16 (1 to 53)	18 (3 to 39)
Median number of positive nodes (range)	1 (0 to 22)	2 (0 to 22)
Lymph node ratio (range)	0.07 (0 to 1.0)	0.14 (0 to 0.95)

**Table 4:** Clinico-pathological data of patients with unavailable chemotherapy-related toxicity data

	**Patient data (n = 40)**
*Clinical data*	
Median age in years (range)	52.5 (23 to 85)
Sex (M:F)	28:12 (70%:30%)
*Tumor location (endoscopic)*	
Gastro-esophageal region	11
Pyloric-antrum	22
Body	3
Fundus	3
Whole stomach, linitis plastica-type	1
*Surgeries*	
Total gastrectomy	10
Proximal gastrectomy	7
Subtotal gastrectomy	13
Inoperable/palliative surgeries	10
Median blood loss in ml (range)	500 (100 to 1000)
Median units of blood transfused (range)	0 (0 to 1)
Morbidity rate^a^	16.6% (5)
Mortality rate	0% (0)
Median duration of hospital stay in days (range)	9 (3 to 35)
*Histopathological data of patients who underwent radical resections*	**Patient data (n = 30)**
Final pathology	
Well-differentiated adenocarcinoma	1
Moderately-differentiated adenocarcinoma	8
Poorly differentiated adenocarcinoma	11
Signet ring cell adenocarcinoma	7
Microscopic foci of adenocarcinoma	1
No residual tumor	2
*T-stage*	
T1	2
T2	9
T3	12
T4	5
Perineural invasion present	6 (20%)
Lymphovascular invasion present	13 (43%)
Median number of lymph nodes harvested (range)	15 (1 to 36)
Median number of positive nodes (range)	1 (0 to 14)
Positive microscopic resection margin	4 (13%)

^a^Complications included duodenal stump leaks (2 patients), delayed gastric emptying grade B (1 patient), pneumonia (1 patient) and intra-abdominal collection (1 patient).

**Figure 1:**
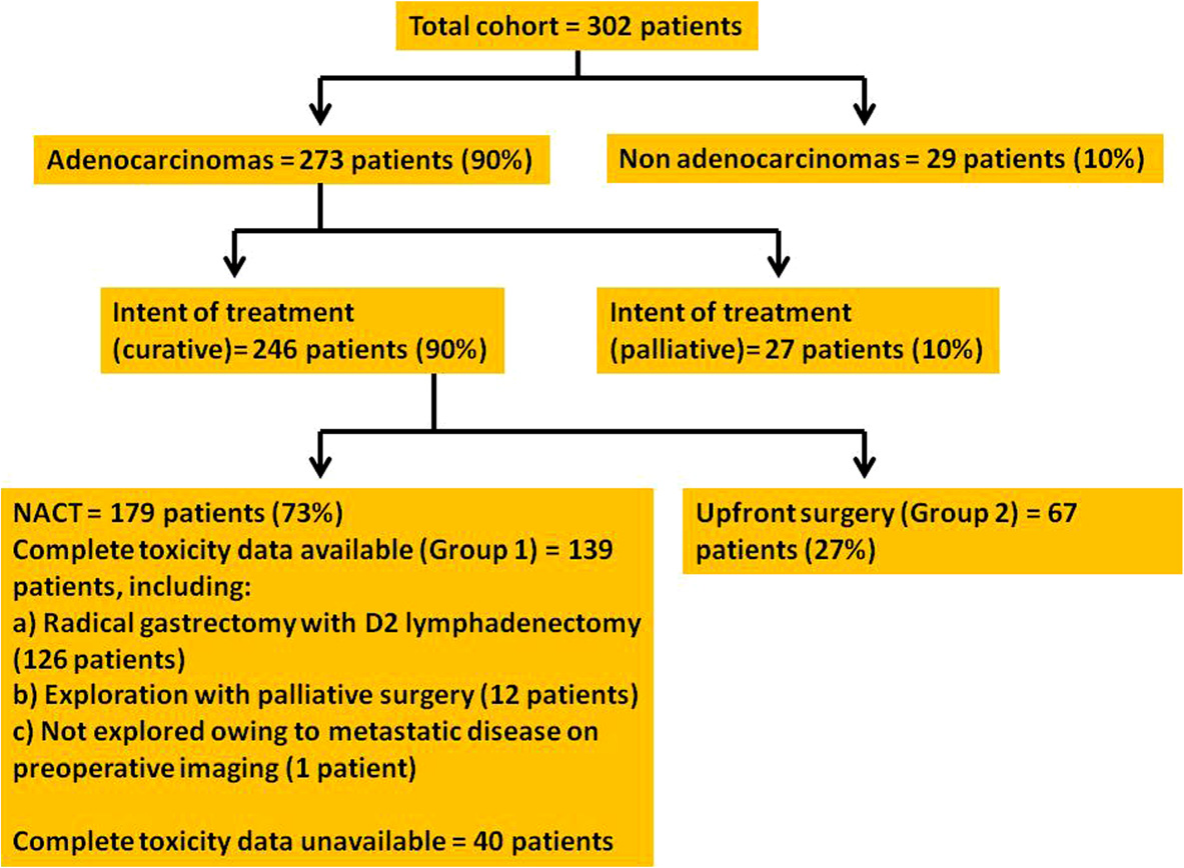
**Breakdown of the entire cohort of 302 patients who underwent surgery for gastric tumors in the study period.** NACT, neoadjuvant chemotherapy.

**Figure 2:**
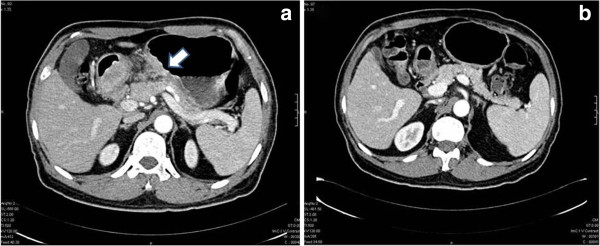
**Axial computed tomography scan sections.** (**a**) A patient with gastric cancer demonstrating thickening of the walls of the mid-body along the lesser curvature (short bold, white arrow). (**b**) Axial section of the same patient demonstrating complete radiological resolution after 3 cycles of NACT. NACT, neoadjuvant chemotherapy.

While the MAGIC trial [10] demonstrated no difference in perioperative morbidity with and without NACT (46% vs. 45%), the two subsequent randomized controlled trials [11,12] demonstrated a trend towards a higher, although non-significant, post-operative morbidity rate in patients who received NACT (26% vs. 19% and 27% vs. 16%, respectively) suggesting the possible risk of increased complications [1]. Another non-randomized study by An *et al*. [13] also found a high incidence of surgical complications following preoperative chemotherapy.

One of the limitations of the trials [10-12], as well as three non-randomized studies from the USA [14], Spain [15] and Korea [13], has been the non-uniform performance of D2 lymphadenectomy in the studies. Therefore, there is a need to critically evaluate perioperative surgical outcomes of radical gastrectomy with D2 lymphadenectomy in this modern era of NACT.

We decided to objectively analyze the perioperative outcomes for standardized D2 gastrectomies following NACT owing to conflicting reports of perioperative outcomes from the six previous studies [10-15]. We have previously reported low morbidity and mortality rates following standardized D2 lymphadenectomy in chemotherapy-naive gastric cancer patients in India [8].

The aims of the current study were: 1) to analyze the toxicity of the chemotherapeutic regimens; 2) to determine the effect of NACT on downstaging cancers prior to resection; and 3) to determine perioperative surgical outcomes after NACT.

## Methods

Patients undergoing surgery for gastric tumors at the Department of Gastrointestinal and Hepato-Pancreato-Biliary Surgical Oncology, Tata Memorial Hospital, Mumbai, India between February 2010 and August 2012 were evaluated retrospectively from a prospectively maintained database. All surgeries were performed by, or under the supervision of, the consultant surgeons in the unit (SVS, MG, SGB).

Preoperatively, all patients were investigated in the same manner with routine blood investigations, including blood counts, liver and renal functions, electrocardiogram and an endoscopy with biopsy. A multi-detector computed tomography (MDCT) triphasic scan of the abdomen and pelvis was performed. Endoscopic ultrasonography was only performed for staging patients with suspected early gastric cancers.

### Planning for NACT

Since 2010, the Tata Memorial Hospital has adopted the routine use of a NACT regimen as part of perioperative chemotherapy for patients with locally advanced (non-metastatic) but resectable gastric cancer. The laboratory (biochemistry and pathology) and imaging results of every patient were discussed at the joint meeting of the Gastrointestinal Disease Management Group, in accordance with routine policy. The indications for directing patients towards NACT were: 1) biopsy-proven gastric adenocarcinoma; 2) MDCT scan indicative of tumor stage (≥T3), perigastric fat stranding, with or without nodal metastases; and 3) no evidence of distant metastases on MDCT scan of the abdomen and pelvis.

The patients were divided into two groups for comparative reasons. Group 1 comprised patients who received NACT followed by surgery, and complete details of their chemotherapy regimens and toxicity, as well as perioperative surgical outcomes was available (n = 139 patients). Group 2 comprised patients who underwent upfront radical gastrectomy for adenocarcinoma (n = 67) due to reasons such as gastric outlet obstruction (n = 43 of 67) or tumors considered early based on joint clinic decisions (n = 24 of 67).

In 40 patients, the chemotherapy was administered elsewhere. These patients were referred to our unit for surgery, and complete details of regimens and toxicity were unavailable. The perioperative surgical data of these patients is presented separately.

### NACT regimen

The protocol consisted of administering either 5 days of 5-fluorouracil (5-FU) for patients with partial gastric outlet obstruction or capecitabine for patients without gastric outlet obstruction, instead of the regular MAGIC trial regimen (21-day infusion of 5-FU) [10].

The regimens employed were based on inferences of the REAL-2 trial [16,17] owing to the reduced toxicity noted in the trial and included the following drugs: epirubicin (E) 50 mg/m^2^ (day 1), cisplatin (C) 60 mg/m^2^ (day 1), 5-FU (F) 650 mg/m^2^ (days 1 to 5), oxaliplatin (O) 130 mg/m^2^ (day 1), capecitabine (X) 1000 mg/m^2^ twice daily (days 1 to 14), docetaxel (D) 50 mg/m^2^ (day 1); in the following combinations: EOX, ECX, EOF, ECF, CAPOX, EO and DCF.

### Definition of chemotherapy-related toxicity

All adverse events and toxic effects were graded according to the National Cancer Institute (NCI) Common Toxicity Criteria, version 3.0.

### Re-staging/assessment of response after NACT

The response to treatment was recorded according to the Response Evaluation Criteria in Solid Tumors (RECIST) guidelines [18]. The response was recorded in case files as stable disease (SD), complete regression/response (CR), disease progression (DP) or partial regression/response (PR), after a joint clinic meeting of the Gastrointestinal Disease Management Group.

### Surgical technique

All patients were restaged with an MDCT scan 2 to 4 weeks after completion of the NACT, at which time a final decision was made for surgery. All procedures were performed in a standardized manner and included proximal, subtotal (distal) and total gastrectomies for gastric cancer, along with a D2 lymphadenectomy, as adopted from the technique practiced by the National Cancer Center, Tokyo, Japan, since 2002 [2,19]. A subtotal (distal) gastrectomy was preferred for tumors located in the pyloro-antral region or in the distal body, provided a 4 cm proximal free margin could be obtained. A proximal gastrectomy was performed for lesions in the proximal/mid-body or cardia of the stomach in which at least half of the distal stomach could be preserved. Total gastrectomy was reserved for lesions in the body or antrum in which adequately free (4 to 5 cm), proximal and distal margins could not be obtained, lesions on the greater curvature with potential metastases to 4sb station lymph nodes [20], and for linitis plastica lesions. We do not routinely perform a prophylactic splenectomy with a total gastrectomy owing to the risk of increased morbidity coupled with the lack of level I evidence to support a survival benefit from the performance of such a procedure [21]. Clinical, pathological and surgical details were recorded.

All patients were administered an antibiotic dose of cefoperazone + sulbactam 2 gm at induction of anesthesia and this antibiotic was continued up to the third postoperative day.

Perioperative mortality was defined as deaths taking place while the patient was still admitted in hospital. Deaths were included irrespective of whether they arose as a result of the surgery or other causes (for example cardiac-related deaths). Postoperative complications have been defined as per our previous publication (anastomotic and duodenal stump leaks were detected either by drainage of bilious contents in the tube drains placed in the abdominal cavity or signs and symptoms suggestive of intra-abdominal sepsis, or both [8]) and delayed gastric emptying (DGE) as per the International Study Group of Pancreatic Surgery (ISGPS) [22]. All complications were graded according to the Clavien-Dindo classification [23].

### Final pathological staging

Final pathological staging was based on the 7th edition of the American Joint Committee on Cancer (AJCC) staging for gastric cancer [24].

#### Statistical analyzes

All statistical analyzes were performed using the Statistical Product and Service Solutions (SPSS) version 14.0 for Windows (IBM SPSS Statistics, IBM, Armonk, NY, USA). Nominal data is provided as number (%) and continuous data as median (range). Non-parametric tests, Mann–Whitney U test for continuous variables and chi-square test for categorical variables were used. All the tests were conducted at 5% significance level.

## Results

Figure 1 provides a full breakdown of the 302 patients who underwent surgery for gastric tumors in the study period. Of the 179 patients who received NACT, complete data on chemotherapy-related toxicity was available for 139 patients and these patients were included in the final analysis as Group 1. However, for the purpose of completeness, the data of the remaining 40 patients is also provided in the results section. Sixty-seven patients (Group 2) underwent upfront gastrectomy with D2 lymphadenectomy.

### Data of Group 1 (n = 139)

#### Epidemiological data (n = 139 patients)

The cohort comprised 42 female and 97 male patients with a median age of 53 years (range 22 to 77 years). Based on endoscopy, the locations of the tumors were: gastro-esophageal region (30 patients), pyloric-antrum (69 patients), body (24 patients), fundus (14 patients) and whole stomach, linitis plastica-type (2 patients).

#### NACT details (including toxicity, n = 139)

Table 1 summarizes the complete data of the patients who received NACT with regimens, number of cycles, patients in whom the regimen had to be changed or cycles reduced, and associated toxicity.

In this cohort, 133 patients (96%) completed NACT. Six patients (4%) could complete only two cycles of NACT due to toxicity. Toxicities were noted in 45 patients (32%). In 2 patients (2%) the protocol had to be changed from ECX to ECF and EOX to EOF because of persistent vomiting resulting from partial gastric outlet obstruction.

Of the 139 patients who underwent NACT, the post-chemotherapy imaging (in comparison to the pre-chemotherapy scan images) indicated PR in 85 patients (61%), SD in 40 patients (29%), CR in 5 patients (4%) and DP in 9 patients (6%) (Figure 2).

#### Surgical details (n = 139 patients)

Based on the radiological findings, 138 patients underwent either a staging laparoscopy with or without laparotomy (12 patients), or direct exploratory laparotomy (126 patients). One patient developed metastases on imaging and was directed to palliative chemotherapy.

A successful surgical resection was undertaken in 126 patients (91%). Of the remaining 12 patients, a palliative antecolic, posterior gastrojejunostomy was performed in 4 patients; while 8 patients had inoperable disease and no procedure could be offered. Details of the surgeries are provided in Table 2. Six patients (4.7%) required intraoperative blood transfusions.

#### Post-operative morbidity and mortality

In this cohort, 15 patients (12%) had complications. These included DGE (grade A, 2 patients and grade B, 4 patients), 2 patients each had suspected duodenal stump leaks, prolonged drain outputs and respiratory complications, and 1 patient each had a collection, a retrogastric hematoma and surgical site infection.

Three patients were re-explored, the 2 patients with suspected duodenal stump leaks and the patient with the retrogastric hematoma. While a defect in the duodenal stump was evident in 1 patient (this was sutured, peritoneal lavage and drains were placed), no clear defect at the duodenal stump or the gastrojejunal anastomosis was found in the other patient. A thorough peritoneal lavage was performed and drains were placed. Both patients made an uneventful recovery. In the third patient who developed a hematoma at exploration, there was a retrogastric hematoma and the spleen appeared ischaemic with no pulsations in the distal splenic artery. A splenectomy was performed and the patient recovered uneventfully after an 8-day stay in the intensive care unit.

In this series, 12 patients had grade I, 1 patient had grade IIIb and 2 patients had a grade IVa complication, as per the Clavien-Dindo classification.

There was no mortality in this series.

#### Histopathological data of patients with resected cancers after NACT (n = 126)

All patients had adenocarcinomas. Details of the histopathology are provided in Table 3. A major pathological response was noted in the primary tumor in 22 patients (17.4%), no residual primary tumor in 15 patients (12%) and scanty foci of tumor in 7 patients (6%). Of the 22 patients, however, tumor was detected in the lymph nodes of 12 patients (55%).

In 5 patients (4%), two distal and three proximal margins demonstrated foci of tumor on final histopathology (R1 resections). Thus, in 121 patients (96%), a curative (R0 resection) could be achieved. In 4 of the 5 patients in whom the distal and proximal margins had shown tumor foci on final histopathology, four specimens were sent for frozen section intraoperatively and were reported to have clear margins. In the case of the fifth patient, the surgery was performed as an after-hour emergency for a perforated gastric cancer and intraoperative frozen section was unavailable.

Clinico-pathological data of patients with unavailable chemotherapy-related toxicity data (n = 40) is presented in Table 4.

### Data of Group 2 (n = 67)

Although 67 patients underwent an exploratory laparotomy with a curative intent, 5 patients were found to have advanced disease not amenable to a curative resection and only a palliative gastrojejunsotomy was performed. Therefore, 62 patients underwent curative resections. The perioperative outcome of these 62 patients is provided as a comparison in Table 2. Histopathological data for comparison with Group 1 is provided in Table 3.

#### Comparison between Group 1 and 2

Table 2 provides a comparison of the demographic and perioperative surgical data between groups 1 and 2. Patients who underwent upfront surgery (Group 2) tended to be older (*P* <0.02), had significantly higher distal tumors (*P* <0.015) and had a longer post-operative hospital stay (*P* <0.001).

## Discussion

The findings of this study, one of the largest, prospective, non-randomized series and the first from India, indicate that NACT does result in a pathological downstaging of the tumor permitting a curative surgical resection in up to 96% of patients. NACT resulted in toxicity of varying grades in 32% of patients. However, such toxicity resulted in a change of regimen or a reduction in the number of cycles in only 5.7% of patients. The findings of the REAL-2 study [16,17] have provided numerous less toxic options to the regimen proposed by the MAGIC trial [10]. NACT did not appear to adversely affect the perioperative surgical outcomes such as median blood loss, number of units transfused, length of hospital stay, morbidity and mortality.

The three randomized controlled trials [10-12] as well as three non-randomized studies [13-15], did not clearly indicate the number of patients undergoing D1 and D2 lymphadenectomy along with the perioperative outcomes specific to these subsets of patients.

In Table 2, we compared the perioperative outcomes between the patients who underwent surgery after NACT with upfront surgery. These two groups are not readily comparable due to the fact that patients undergoing upfront surgery generally do so owing to their distally-located tumors being within the stomach leading to varying degrees of gastric outlet obstruction. The result is that these patients have a poor tolerance to chemotherapy in the neoadjuvant setting. They may also be nutritionally depleted resulting in the observed higher morbidity rates. The data also indicates that the patients offered upfront surgery tended to be older in age (with the potential for pre-existing co-morbidities), which could also have contributed to an increased morbidity and resultant longer hospital stay.

Our data suggests that NACT does not affect the lymph node yield at the time of surgery (16 vs. 18, *P* <0.25). This is in contrast to a recent publication by Wu *et al*. [25] who found a reduced lymph node yield (<15 harvested lymph nodes) following NACT compared to patients who underwent upfront surgery for gastric cancer (7.7% vs. 24.1%). The lower median number of lymph nodes reported in the final histopathological analysis in our series compared to series from Japan [7] and Korea [26] cannot be readily explained considering that the technique of lymphadenectomy performed by us is based entirely on the procedure described by the National Cancer Centre, Tokyo. We can only conjecture that this may be related to the other important variable in improving lymph node yield, the diligence of the pathologist [27]. The development of disease-centric management groups comprising organ/disease-specific, dedicated surgeons and pathologists, as has been the policy at our institution, will help clarify these aspects in the near future [28].

In our study, there are multiple regimens employed for NACT. Rather than perceiving this as a weakness of the study, this is in fact a reflection of our experience with Indian patients and their inability to tolerate the standard chemotherapeutic regimens prescribed for gastric cancer in other parts of the world. Thus, we had to resort to different combinations in order to tailor the chemotherapeutic regimen to the tolerability of the patient. A NACT protocol of EOX, as per our own experience, with an interval of 4 to 6 weeks between chemotherapy and surgery, would constitute the most useful schedule to be followed.

On comparing our study with the other three non-randomized studies [13-15], one of the major findings reported in the other three studies is a high morbidity (29.3 to 38%, compared to 12% in our study). Possible reasons for the lower morbidity in our series could include the lack of radiotherapy in the neoadjuvant setting as well as the increased experience with the standardized technique of D2 lymphadenectomy in a high-volume centre. Major pathological response to the neoadjuvant therapy was reported only in the study by Valenti *et al*. [15] to be 33% (47.6% in the chemoradiotherapy arm and 13.3% in the chemotherapy arm). The major pathological response in our study (in which only chemotherapy was used) was 17%, which is similar to the response rate in the study by Valenti *et al*. [15]. The perceived benefit of better pathological response noted by the combination of chemotherapy and radiotherapy compared to chemotherapy alone in the two studies must be regarded with caution especially since the surgical morbidity rates are significantly higher (30.9% in the study by Valenti *et al*., compared to 12% in the present study).

An interesting finding in our series has been the identification of positive lymph nodes (55%) even in patients who demonstrated a major pathological response of the primary tumor to chemotherapy on histopathology. These data indicate that administration of NACT does not obviate the need for a complete radical resection and D2 lymphadenectomy. Although this observation is based on a relatively small cohort of patients, its significance cannot be undermined pending larger series in the future.

Our centre constitutes a referral unit and as a result complete data of all the patients who may have received chemotherapy outside the hospital, prior to referral, was not very clear in terms of toxicities. Hence these patients were not included in the analysis. However, the epidemiological and surgical data of these patients has been presented, which is not different from the larger cohort.

At the time of the study period, the only accepted indication for staging laparoscopy was in patients with T3 or T4 gastric cancer without evidence of lymph node or distant metastases on high quality preoperative imaging [29]. This clinical scenario was very uncommon in our setting and hence the selective use of staging laparoscopy in our series.

Surgeons have harbored concerns regarding operating on patients whose tissues have been treated with neoadjuvant chemotherapy/chemoradiotherapy. However, these subjective difficulties need to be objectively assessed in terms of perioperative outcomes. From our study we can infer: 1) based on the objective comparison of our perioperative surgical outcomes of upfront surgery versus surgery after NACT, there is no further increase in morbidity or mortality; and 2) there is no significant disease progression to preclude surgery after NACT based on our observation that only 12 of 139 patients (8.6%) were ineligible for a radical resection. These findings should convince surgeons dealing with gastric cancer to move towards NACT followed by gastrectomy with D2 lymphadenectomy as an evidence-based standard of care for gastric cancer.

## Conclusions

Perioperative outcomes of gastrectomy with D2 lymphadenectomy for locally advanced resectable gastric cancer are not influenced by NACT. Furthermore, the quality and radicality remains unaffected. The number of lymph nodes harvested was unaltered by NACT and, more importantly, metastases to lymph nodes were noted even in patients with major pathological response of the primary tumor. D2 lymphadenectomy should be performed even in patients with a good objective response of the primary tumor to NACT.

This is the first study from India to demonstrate no change in morbidity, mortality and lymph node yield in patients undergoing gastrectomy with D2 lymphadenectomy in the post-MAGIC trial era compared to outcomes in patients undergoing upfront surgery prior to the introduction of NACT protocols. More importantly, lymph node metastases were noted even in patients who demonstrated a major pathological response of the primary tumor to NACT supporting the need for D2 lymphadenectomy in all patients.

## Abbreviations

5-FU: 5-fluorouracil; AJCC: American Joint Committee on Cancer; C: Cisplatin; CR: Complete regression/response; D: Docetaxel; DGE: Delayed gastric emptying; DP: Disease progression; E: Epirubicin; F: 5-FU; ISGPS: International Study Group of Pancreatic Surgery; MDCT: Multi-detector computed tomography; NACT: Neoadjuvant chemotherapy; NCI: National Cancer Institute; O: Oxaliplatin; PRL: Partial regression/response; RECIST: Response Evaluation Criteria in Solid Tumors; SD: Stable disease; X: Capecitabine.

## Competing interests

All authors declare that they have no competing interest.

## Authors’ contributions

SVS: conceived and designed the study, interpreted the data and critically revised the manuscript; SGB: designed the study, collected the data and interpreted the analysis, and drafted the manuscript; SDT: performed the data analysis including the statistics; KV, SA and KS: collected the data; SM and MG: Critically revised the manuscript; All authors read and approved the final manuscript.
